# Perforating Wounds of the Knee Joint

**Published:** 1898-03-26

**Authors:** 


### PERFORATING WOUNDS OF THE KNEE JOINT.

The discussion of this subject at the last meeting of the Clinical Society showed how great had been the improvement in the treatment of these cases during recent years. As Mr. Howard Marsh said, cases such as Mr. Wallis had related had formerly commonly ended either in the death of the patient from septicæmia, or, if this were escaped, amputation of the limb, whereas at the present day it was seen how closely the synovial membranes of joints resembled the peritoneum in their tolerance of aseptic operative interference. Mr. Wallis pointed out that only those cases ran an aseptic course which were immediately treated, but even prolonged suppuration did not necessarily mean emkylosis, if certain lines of treatment were carried out. Mr. Barker thought drainage was often overdone, and that in many cases it was not required, and as to irrigation he thought the liquid used was often too irritating. The action of irrigation was, he said, only mechanical, and warm water was consequently as efficacious as any other liquid. Mr. Watson Cheyne joined in the protest against washing out septic cavities with antiseptic remedies. His practice was when a joint had been wounded to incise it freely, and to drain it for 24 or 48 hours, when, if all was going on well, the drain could be removed. It was certainly unsafe to close up a septic wound without drainage. He advised that all the soft parts round the wound should be sponged with undiluted carbolic acid.

